# Comparison of the preventive effect of colchicine versus diphenhydramine, prednisolone, and a combination therapy on intraperitoneal adhesion bands: an experimental study in rats

**DOI:** 10.1186/s12893-023-01981-0

**Published:** 2023-04-10

**Authors:** Seyed Ali Malekhosseini, Behzad Alizadeh, Ahmad Hosseinzadeh, Reza Shahriarirad, Reyhaneh Naseri, Kourosh Kazemi, Alireza Shamsaeefar, Nader Tanideh

**Affiliations:** 1 Shiraz Transplant Center, Abu Ali Sina Hospital, Shiraz University of Medical Sciences, Shiraz, Iran; 2 Department of Surgery, Shiraz University of Medical Sciences, Shiraz, Iran; 3 Thoracic and Vascular Surgery Research Center, Shiraz University of Medical Science, Shiraz, Iran; 4 Student Research Committee, School of Medicine, Shiraz University of Medical Sciences, Shiraz, Iran; 5 School of Medicine, Shiraz University of Medical Sciences, Shiraz, Iran; 6 Stem Cells Technology Research Center, Shiraz University of Medical Sciences, Shiraz, Iran; 7 Pharmacology Department, Shiraz University of Medical Sciences, Shiraz, Iran

**Keywords:** Peritoneal adhesion, Animal model, Hydrocortisone, Methylprednisolone, Colchicine, Diphenhydramine

## Abstract

**Background:**

Peritoneal adhesion formation is an inevitable consequence of abnormal repair of the peritoneum following different peritoneal injuries of intra-abdominal operations with the subsequent morbidity that they represent. Vast efforts have been made to elucidate the cause and prevent the development of abdominal adhesions. The aim of our study is to compare the capability of colchicine versus diphenhydramine (DPH) and methylprednisolone (MP), and also prednisolone in adhesion prevention.

**Methods:**

Sixty-one male Wistar stock rats were divided into four groups. The first group attended as the control group. Groups 2, 3, and 4 received oral combination of MP + DPH solution (20 mg/kg), colchicine (0.02 mg/kg), and prednisolone (1 mg/ kg), respectively. Adhesion bands were induced by standardized abrasion of the peritoneum through a midline laparotomy. All rats were sacrificed on the 15^th^-day post medication administration and the subjects underwent an exploratory laparotomy. The presence of adhesions was evaluated with the modified using Nair's classification.

**Results:**

The proportion of the control group with substantial adhesion bands (73.3%) was significantly higher than that of the MP + DPH (13.3%), colchicine (33.3%), and prednisolone (31.3%) groups. There were significant differences between the scores of the control and the MP + DPH, colchicine, and prednisolone groups (*P* = 0.001, 0.028, and 0.019, respectively). There was no statistically significant difference to favor colchicine against MP + DPH (*P* = 0.390) or MP + DPH against prednisolone (*P* = 0.394).

**Conclusions:**

Both colchicine and combination of DPH + MP prevented postoperative abdominal adhesions separately in our study. However, the lowest adhesion formation rate was observed in the DPH + MP group, even lower than the prednisolone group.

## Background

Postoperative intraperitoneal adhesions virtually, can form after any trans-peritoneal operation ranging from minimal scarring of serosal surface to firm agglutination of nearly all structures [1–3]. Statistical analysis shows a high incidence rate of adhesions around 66% in patients with previous abdominal operations [4], which are a major clinical, social and economic concern [5, 6], as they may result in chronic pelvic pain [7], intestinal obstruction [8], female secondary infertility [9], and additional surgery to resolve such adhesion-related complications [10]. The recurrence rate of adhesions despite the conservative approaches or adhesion lysis treatments is still high. For instance, the recurrence rate in intestinal obstruction is as high as 53% [11]. Therefore, intraperitoneal adhesions are associated with increased postoperative clinical complications, economic costs and workload. This significant morbidity and mortality warrant the concern of anti-adhesion strategies.

The significant accumulative rate of clinical and financial evidence on adhesion-related health burdens has made researchers come up with various therapeutic strategies intended to prevent postoperative adhesion formation. Several mechanisms have been defined for adhesion development, so administering agents to interfere with the pathogenic pathways of adhesion formation can be an effective treatment for reducing adhesion formation after surgery. One of the most recognized mechanism for post-surgical adhesion formation is the one related to histamine discharge [12], and it has been documented that intestinal mast cells are responsible for releasing a large number of inflammatory mediators, including histamine [13]. Systemically applied antihistamines have been reported to prevent development of adhesion by reducing vasodilatation, the permeability of blood vessels and prevent the outflow of fibrinogen [14, 15]. Moreover, corticosteroids have also been stated to have the similar effect on decreasing vascular permeability by inhibiting cell proliferation and collagen synthesis as their anti-inflammatory trait [16, 17]. The anti-adhesion effect of antihistamines has been proposed; however, the literature does not contain a sufficient number of studies on the subject.

Colchicine, is an ancient drug generally used to treat acute gout attacks and familial Mediterranean fever [18]. Recently, colchicine has been proven to be effective in idiopathic pulmonary fibrosis, actinic keratosis, reducing intraarticular adhesion following knee surgery and cystic fibrosis due to its strong anti-inflammatory and anti-fibrotic effects [19–22]. Potential anti-inflammatory activities include, inhibition of histamine release by mast cells, suppression of pro-collagen synthesis, and promotion of collagenase [23]. In line with our intention, we hypothesized using oral colchicine, a unique anti-inflammatory alkaloid drug, for its histamine release inhibition traits secreted from mast cells, and administration of the anti-histamine drug diphenhydramine (DPH), which stabilizes mast cells from degranulation during the initial stages of inflammation [14].

We have previously demonstrated the effectiveness of macrolides (sirolimus) and prednisolone in preventing intra-abdominal adhesion formations [24]. Based on our hypothesis and evidence available in the literature, we designed this study to evaluate and compare the effects of DPH and methylprednisolone (MP), and also colchicine in the prevention of abdominal adhesion.

## Methods

The study protocol was a randomized, and experimental animal study and conducted in accordance with the principles of the guideline of the Ministry of Health and Education of Medicine of Iran for the care and use of laboratory animals at the Center of Comparative and Experimental Medicine, Shiraz University of Medical Sciences, Shiraz, Iran, with ethical approval from the Institutional Animal Care and Use Committee of Research Center of Shiraz University of Medical Sciences(Ethical code: IR.SUMS.MED.REC.1384.S2719). The sample size was assigned based on previous studies, with assessing the risk of drop-out risk and along with the minimum requirement to attain reliable results [25–27].

This study is based on three consecutive steps: first, the induction of adhesions by standardized abrasion of the peritoneum through a midline laparotomy [28], following the assessment of morbidity and mortality rates of different solutions, and eventually, testing the ability of the exact gavage solutions to prevent adhesions. Sixty-one male Wistar stock rats were employed, weighing 200 ± 20 g each. Animals were kept under standard laboratory conditions in similar metal shelves with 55 ± 5% relative humidity, a 12/12-h light/dark cycle, and 22∘ C ± 2֯c temperature. They were fed a standard laboratory diet with free access to food and water. The rats were randomly assigned to four groups based on simple randomization method. Group A consists of 15 rats that were maintained as control, receiving no treatment. In group B, a combination of methylprednisolone and diphenhydramine (MP + DPH) solution (Iranhormone factory, Iran) with a concentration of 20 mg/kg was administered; Group C received colchicine (Modacine, Modava, Iran) dissolved in distilled water at a concentration of 0.02 mg/kg/day was administered; and in group D 1 mg/ kg oral prednisolone (as 5 mg tablets made by Iranhormone factory, Iran) daily in the morning using long metal gavage for an experimental period of two weeks plus a single dose of the medications two hours preoperatively. The dosage of medications was based on that of human subjects and also similar to previous studies [15, 24, 29–31].

At the end of the 15th-day post-operation, after prep. and drep. with antiseptic solution, containing povidone-iodine and 70% alcohol, all rats were decapitated swiftly. A midline xipho-pubic laparotomy was then performed, using sterile techniques, as in similar studies [1, 4, 9].

Adhesions were qualitatively scored blindly by an independent surgeon who was not informed of the subjects’ study groups. The adhesions were evaluated according to the widely used score systems of Nair classification [32]. The degree of adhesion was scored as follows: grade 0, complete absence of adhesions; grade1, single-band of adhesions, between viscera or from one viscus to the abdominal wall; grade 2, two bands, either between viscera or from viscera to abdominal wall; grade 3, More than two bands, between viscera, or viscera to the abdominal wall, or whole intestines form a mass without adherence to the abdominal wall; grade 4, Viscera is directly adherent to the abdominal wall, irrespective of the number and extent of adhesive bands. Based on Nair's classification, adhesions were also classified into two subgroups: insubstantial adhesions (grade 0, 1) and substantial adhesions (grade 2–4).

The subject’s data were entered into SPSS version 23 (IBM, United States). The count and percentage were calculated for qualitative variables. The Fisher’s exact test or Chi-square test was used to compare the results among groups. Differences were defined significant as *P* < 0.05.

## Results

**Fig. 1:**
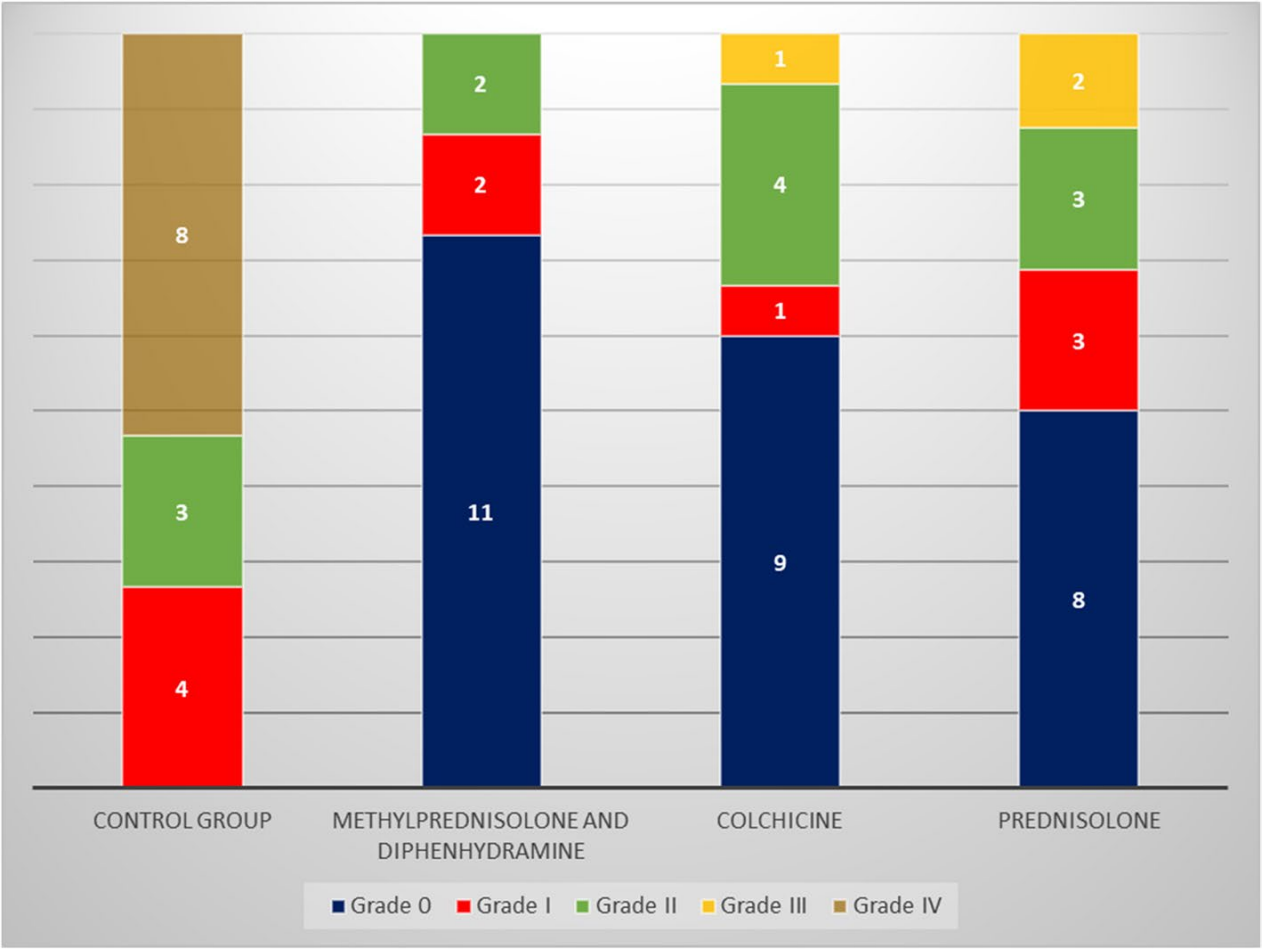
Adhesion grading among rats based on the treatment groups

At the end of the 14-day post-operation period, no mortalities were observed among the groups, and no healing problems such as infection and delayed wound closure occurred in any group. The results are illustrated in Fig. 1. The MP + DPH group demonstrated the highest rate of subjects without adhesion (73.3%), followed by the colchicine group (60.0%) and the prednisolone group (50.0%). All subjects in the control group developed adhesion.

Based on Fisher’s exact test, there was a significant difference among the groups in our study (*P* < 0.001). As demonstrated in Table 1., all three groups of MP + DPH, prednisolone, and colchicine had a significant difference with the control group. The majority of subjects in the control group developed a grade IV adhesion (53.3%), while in the treatment groups, the majority had a grade 0 adhesion. However, there was no significant difference in the intergroup evaluation of the treatment groups.

**Table 1:**
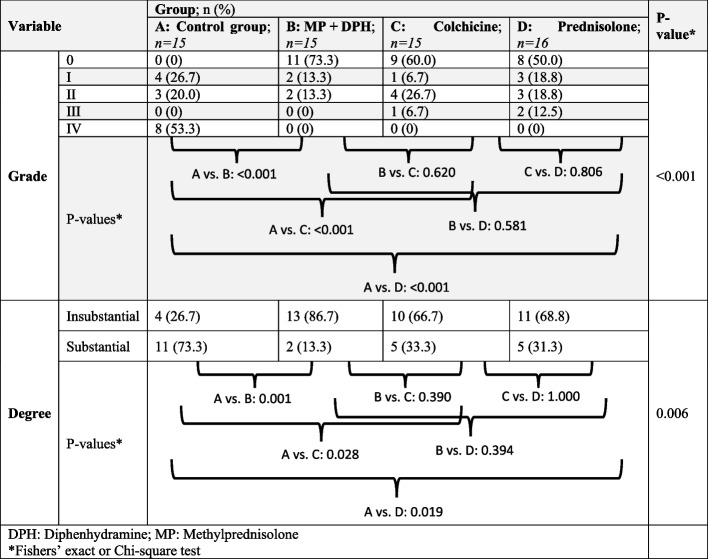
Evaluation and comparison of the adhesion grading among the treatment groups in our study

When categorizing adhesion grades based on their degree, there was a statistically significant difference among the groups (*P* = 0.006). All treatment groups had significantly lower rates of substantial adhesion compared to the control group (Table 1.). The MPH + DP group demonstrates lower rates of substantial adhesion compared to the prednisolone (13.3 vs. 31.3%; *P* = 0.394) and the colchicine (13.3 vs. 33.3; *P* = 0.390) groups, while the colchicine group also was particularly similar to the prednisolone group (31.3 vs. 33.3%; *P* = 1.000). However, there was no significant difference among the treatment groups.

## Discussion

We compared the possible synergistic effect of MP as an intermediate-acting, synthetic glucocorticoid [33] plus DPH as a widely used antihistamine [34] in preventing experimentally induced peritoneal adhesions in rats along with assessing the efficacy of colchicine, a drug with anti-inflammatory properties [18]. Our experimental data in an animal model has suggested that a combination of MP and DPH has the potential to be efficacious in preventing peritoneal adhesion formation by 60% compared to the control group. The present study also provides data to compare the individual effect of systemic administration of prednisolone as a corticosteroid, which resulted in a lower rate of 42% compared to the control group. We also confirmed that oral administration of colchicine led to a significant lower rates of postoperative adhesion formation of 40% compared to the control group.

Studies have introduced several mechanisms for adhesion development, but the one related to histamine discharge is known to be one of the chief suspects of pathogenic adhesion formation cascade [14]. Histologically, the greater omentum is composed of a connective framework carrying vessels and featuring mesothelial spaces surrounded by two layers of flat mesothelial cells. The thicker areas of this 'organ' contain macrophages, lymphocytes, plasma cells, and mast cells. [35] Injured peritoneal surfaces and the presence of ischemic lesions trigger the local release of histamine, thereby the disruption of stromal mast cells releases vasoactive substances such as histamines and kinins, increasing vascular permeability, which contributes to the collection of a fibrin-rich exudate that covers the injured area, leading to the formation of adhesions [15, 16, 36]. A mainstay of therapy is mast cell-stabilizing drugs; therefore, a burst of corticosteroids in addition to antihistamines could be beneficial [37, 38]. It was also found in our study that when the two agents were used together (MP + DPH), they prevented peritoneal adhesions score even more significantly compared to the control group; however, the differences between administration of singular corticosteroid (prednisolone) and the combination group (MP + DPH) was not considered significant statistically. The current paper provides proof of the principle that adhesion formation can be diminished by oral administration of potent well-studied corticosteroids like prednisolone; however, an important limitation of steroid use for adhesion prophylaxis is that these agents decrease wound healing and put anastomoses at risk. Moreover, other stated adverse events of corticosteroids, such as suppression of the pituitary-adrenal axis and immunosuppression, limit the clinical indications for corticosteroids as anti-adhesive agents in human patients [39–41].

According to our observations, the colchicine group also was particularly similar to the prednisolone group. However, despite the indicated adverse events of corticosteroids, there are limited and rare potential side effects of colchicine in prolonged high dosage administration [42, 43]. The effect of colchicine and related substances on mast cells was initiated by Padawer et al. [44].He described the idea that mast cells prominently consist of microtubules and microfilaments. Drugs that interfere with microtubules, such as colchicine, have a powerful effect on inhibition of proposed microtubules that were involved in cell secretion, such as histamine release from mast cells [42, 45].Subsequently, colchicine dampens the effects of mast cells. Our observations are entirely consistent with his description; the reduction in the number of rats affected by extensive adhesions was significant compared to the control group when colchicine was administered through oral gavage to rats. Clinically, colchicine is a drug that is safely used in men that have been in continuous use for more than 3000 years [18, 46]. Prescription of colchicine in patients with normal renal and hepatic function is usually safe; however, gastrointestinal discomfort such as diarrhea, vomiting, and abdominal cramps may occur in most patients for 24 h [18, 47], and reported untoward reactions to colchicine are rare, similar to our investigation, no unpleasant reactions of colchicine were observed during the postoperative period of the experiment.

Previously, Dargenio et al. [48], and Granat et al. [39] compared the effect of colchicine and dexamethasone in the prevention of adhesions, dispensing the colchicine intramuscularly or intraperitoneally, whereas we administered it orally. They demonstrated that colchicine conceded better outcomes compared to dexamethasone; however, it provided better results when mixed with dexamethasone. Bokeriya et al. [49] investigated the anti-inflammatory effects of colchicine used in biodegradable films based on gelatin to prevent adhesion development in the postoperative pericarditis rabbits model. Significant reduction of the severity of pericardial adhesions and improved visibility of coronary arteries were detected. Rojkind and Kershenobich [50] used colchicine to prevent fibrosis in liver cirrhotic patients and demonstrated the colchicine as a collagen synthesis inhibitor, anti-proliferative, and fibrosis-modulator [51]. Related to our study, Yıldız et al. [52] experimented on rats consuming oral colchicine at a dose of 50 mcg/kg/day. They observed a significant difference among the adhesion scores of the orally given colchicine group, both macroscopically and microscopically. In our study, colchicine was administered orally at a dose of 0.02 mg/kg/day and significantly reduced the intensity of adhesion formation in the peritoneal cavity, however, without reaching a statistically significant difference in comparison with the MP + DPH group (*P* = 0.390).

### Limitations

Admittedly, among the limitations of the currently employed model is that this study looks at a solitary point in time for determining the adhesion promotion or inhibition of these products, and two weeks were chosen for the evaluation of adhesion during an early postoperative period which might not correlate with the degree of adhesion formation that proceeds after longer intervals. However, this appears to be a standard timeframe for evaluating adhesions in animal care and the limitations of the model we used [53]. Therefore, further studies are demanded to investigate the influence of colchicine, the combination of (MP + DPH) and prednisolone on adhesion formation at later stages associated with clinical practice. Also, the systemic adverse effects of the studied drugs should be evaluated, and their consequences should be considered alongside their adhesion prevention nature. Furthermore, colchicine was only available in oral form in our country. However, our preliminary results may encourage further investigations to test the anti-adhesion effect of colchicine in varying doses via different routes of administration. Besides, it should be mentioned that should that we evaluated the effect of these medications on only on male rats, since it remains unclear whether or not one gender is more prone to develop adhesions. We expect not to identify any significant differences on gender in our study, therefore we conducted our study with male wistar stock rats, as it was available at the time of our study. Additionally, further studies in human subjects as a more convenient and relatively safe mode of systemic therapy in the prevention of postoperative formation of peritoneal adhesion are needed to confirm the efficacy of these agents. Moreover, although we demonstrated the efficacy of MP + DPH, further studies focusing on the solus use of DPH in preventing abdominal adhesion are required. Certainly, the ultimate effect on postoperative adhesions could be different than seen here. Finally, the cytokine and chemokines network system and related and its’ direct role in the pathogenesis of adhesion formation were not assessed in our study, which warrants further molecular and immunocytological studies in this regard.

## Conclusions

In conclusion, the most substantial intra-abdominal adhesion prevention the in the MP + DPH treated group, while colchicine also demonstrated relatively similar preventive properties as the prednisolone group. Therefore, although corticosteroids have demonstrated satisfactory intra-abdominal adhesion preventive properties, the combination with DPH can enhance their effects and can be proposed as an adds-on therapy, especially in high-risk patients. Furthermore, we believe that colchicine can be an excellent anti-adhesive agent taking its ease of use, suitable for safe, long-term use, and effectiveness into account. Nevertheless, further detailed experimental and clinical studies are needed to perform an extensive analysis of the effects of these anti-adhesive agents in the prevention of adhesions.

## Data Availability

All data regarding this study has been reported in the manuscript. Please contact the corresponding author if interested in any further information.

## Abbreviations

DPH: Diphenhydramine
MP: Methylprednisolone
